# A nested case-control study on the risk factors of deep vein thrombosis for Chinese after total joint arthroplasty

**DOI:** 10.1186/s13018-019-1231-9

**Published:** 2019-06-24

**Authors:** Hong Xu, Shaoyun Zhang, Jinwei Xie, Yiting Lei, Guorui Cao, Guo Chen, Fuxing Pei

**Affiliations:** 1 Department of Orthopaedic Surgery, West China Hospital, Sichuan University, Chengdu, 610041 People’s Republic of China; 2 Department of Orthopedics, The Third Hospital of Mianyang • Sichuan Mental Health Center, No. 190 The East Jiannan Road, Mianyang, 621000 People’s Republic of China

**Keywords:** Deep vein thrombosis, Total knee and hip arthroplasty, Risk factor

## Abstract

**Background:**

Deep vein thrombosis (DVT) is one of the life-threatening complications of total joint arthroplasty (TJA) postoperatively, and its risk factors are still controversial. The aim of this study was to identify the risk factors of DVT after TJA.

**Study design and methods:**

A nested case-control study based on a large dataset of 15,326 patients undergoing TJA was performed. Potential risk factors of DVT and demographic information were extracted from the electronic health record. Patients with DVT (73 patients) were treated as study group while non-DVT patients who were matched 1:4 according to the anticoagulant type, were considered as control group (292 patients). These variables of potential risk factors for DVT including age, sex, body mass index (BMI), American Society of Anesthesiologists class, comorbidity, preoperative hemoglobin (HB) level and analgesic use, surgical site (knee or hip) and type, the start time of drug anticoagulation, ambulation time, transfusion, and whether to use tranexamic acid (TXA), drain, human serum albumin, and measures of physical thromboprophylaxis after operation were collected and evaluated by survival analysis and presented as *P* value and odds ratio with 95% confidence interval.

**Results:**

There were 15,326 patients underwent TJA and 73 (0.48%) patients had DVT among them, and the occurrence rates were 0.71% for the patients underwent total knee arthroplasty (TKA) while 0.24% for total hip arthroplasty. The risk factors associated with DVT included TKA (compared with THA), advanced age (> 70 years), drain use, and delayed ambulation (≥ 72 h) postoperatively.

**Conclusion:**

The present results suggest that the occurrence rate of DVT on the patients underwent TJA was low (0.48%) relatively. And the risk factors associated with increased risk of DVT included TKA (compared with THA), advanced age, drain use, and delayed ambulation postoperatively. Individualized and more efficient risk stratification protocols of anticoagulation after TJA for Chinese may need to be developed in the future.

## Introduction

TJA is a successful surgical treatment for end-stage knee or hip diseases due to that it can relieve pain, improve function, and elevate the quality of patients^,^ life [1, 2]. With the advancement of the Enhanced Recovery After Surgery program, the number of patients undergoing TJA has significantly increased in recent years. However, postoperative DVT is still one of the most severe complications after THA and TKA. The main danger associated with DVT is pulmonary venous thromboembolism (PTE), which can occur when the clot breaks away from the vessel wall and travels as far as the lungs, which is associated with significant morbidity and mortality after TJA. A previous study [3] that recruited into 1,805,621 patients undergoing TJA showed that the in-hospital mortality rate among patients with VTE was significantly higher than patients without VTE (7.1% vs. 0.30%, *P* < 0.0001). So, it is quite essential to identify the risk factors of DVT after TJA.

Despite several prior studies [4–6] have demonstrated the risk factors of the DVT after TJA, the ability to identify risk factors of DVT after TJA remain a great challenge for joint surgeons for a long time because its lower incidence rate. From 2013 to 2016, a multicenter study on the efficacy and safety of perioperative management of THA and TKA was carried out in China, and a large database containing 15,326 patients has been established, which allows us to evaluate thousands of patients and get a credible result about the risk factors of DVT for the Chinese after TJA. Although the risk factors of DVT for the patients undergoing TJA has been evaluated previously, which is still needed to explore for Chinese population due to most of previous studies were based on western population. Hence, this study, which involves the largest Chinese patients so far, was performed to identify the risk factors of DVT for Chinese population undergoing TJA.

## Materials and methods

### Data source

This study was a secondary analysis of a large database generated from a prospective multicenter study on the efficacy and safety of perioperative management of THA and TKA, and the related data were collected from dozens of institutions in China prospectively. This database included patient-level hospital discharge data provided by 26 university teaching hospitals in China (10 national and 16 regional hospitals) sponsored by the Chinese Health Ministry (project number 201302007). The data elements consisted of patients’ demographics, diagnosis, procedure information, comorbidities, medication history, laboratory values, and in-hospital mortality. American Society of Anesthesiologists class was evaluated by an anesthetist before the surgery and recorded for each patient. We confirmed the completeness and validity of the data by comparing with data from hospital information systems; and we checked for missing data or incorrectly coded values in the current database carefully. In addition, this study was deemed exempt by the hospital’s institutional review board (2012–268).

### Patients’ demographics

All surgeries (7789 in TKA and 7537 in THA) including primary unilateral, simultaneous bilateral, and unilateral revision TJA were enrolled into this study, and the diagnosis included osteoarthritis, rheumatoid arthritis (RA), osteonecrosis of the femoral head, developmental dysplasia of the hip, ankylosing spondylitis involves hip joint and fracture of femoral neck. The comorbidities and medication history included hypertension, type 2 diabetes, and preoperative analgesic use. All patients were screened before operation and were discharged respectively with Doppler Ultrasound (DUS) to exclude DVT. Moreover, if the patients had these symptoms including swelling, pain, lower skin temperature of limb, and high tension of soft tissue, DUS was performed immediately. The chief surgeon determined the protocol of thromboprophylaxis, ambulation time, and whether to use TXA and drain or not.

### Statistical analyses

Since the details of drug anticoagulation protocol including the dosage, frequency and course of treatment were not recorded, patients with DVT were treated as study group while non-DVT patients matched 1:4 by the type anticoagulant were regarded as control group. The patients with DVT were selected from the database as the study group, and the patients of control group were selected randomly with a ratio of 1:4 matched according to the anticoagulant type in non-DVT patients, which was implemented by SPSS version 21 software (IBM, Armonk, NY). This means that for every patient in the study group, there were four patients who were given the same anticoagulant were matched in the control group. Fifty-six patients were treated with rivaroxaban, 16 patients with low-molecular heparin, and 1 patient with apixaban in the group of DVT, consequently, 224 patients treated with rivaroxaban, 64 patients with low-molecular heparin, and 4 patients with apixaban were matched as control group. Variables were summarized as mean ± standard deviation (SD) for continuous variables and frequency (proportion) for categorical variables. Besides, some continuous variables including age, BMI, start time of drug anticoagulation, and ambulation time also were transformed into categorical variables. Univariable analysis was performed to identify independent risk factors, and multivariable survival analysis was carried out to evaluating the risk factors of DVT for the patients undergoing TJA. Odds ratios (OR) for regression modeling and their 95% confidence intervals (CI) were calculated. The level of significance level was set as *P* < 0.05. All data analyses were performed using SPSS version 21 software (IBM, Armonk, NY).

## Results

### Patient population and demographics

Demographic characteristics of the patients undergoing TJA were shown in Table 1. In total, there were 15,326 patients undergoing TJA, and 73 (0.48% for all patients; 55 [0.71%] for TKA and 18 [0.24%] for THA) patients had DVT among them, which were treated as study group, while 292 non-DVT patients were matched as control group. And our population were predominantly female (68.22% vs. 31.78% male). The mean age at the time of surgery was 66.52 ± 9.89 years.

**Table 1:** Baseline characteristics of the patients and univariable analysis of risk factors for DVT

Perioperative factors	DVT (*n* = 73)	NonDVT (*n* = 292)	*P* value	OR	(95%CI)
Age (years)					
Mean ± SD	66.52 ± 9.89	62.50 ± 12.28	0.021	1.026	(1.004–1.049
≤ 60	17 (23.29%)	102 (34.93%)	1.00 (reference)	–	–
> 60 to ≤ 70	26 (35.62%)	116 (39.93%)	0.419	1.290	(0.696–2.389)
> 70	30 (41.10%)	74 (25.34%)	0.020	2.029	(1.116–3.688)
Sex					
Male	16 (21.92%)	100 (34.25%)	1.00 (reference)	–	–
Female	57 (78.08%)	192 (65.75%)	0.073	1.660	(0.953–2.891)
BMI (kg/m^2^)					
Mean ± SD	26.20 ± 4.03	25.14 ± 3.85	0.063	1.053	(0.997–1.113)
< 25	32 (43.84%)	162 (55.48%)	1.00 (reference)	–	–
≥ 25	41 (56.16%)	130 (44.52%)	0.113	1.454	(0.916–2.309)
ASA class					
I	35 (47.95%)	103 (34.27%)	1.00 (reference)	–	–
II	31 (42.47%)	151 (51.72%)	0.105	0.669	(0.412–1.088)
III and IV	7 (9.86%)	38 (13.01%)	0.237	0.613	(0.272–1.380)
Hypertension					
No	47 (64.38%)	224 (76.71%)	1.00 (reference)	–	–
Yes	26 (35.62%)	68 (23.29%)	0.052	1.625	(0.995–2.652)
Type 2 diabetes					
No	68 (93.15%)	272 (93.15%)	1.00 (reference)	–	–
Yes	5 (6.85%)	20 (6.85%)	0.052	1.625	(0.995–2.652)
Preoperative Hb (g/dL)	127.69 ± 12.86	129 ± 16.48	0.393	0.994	(0.979–1.008)
Preoperative analgesic use					
No	63 (86.30%)	218 (74.66%)	1.00 (reference)	–	–
Yes	10 (13.70%)	74 (25.34%)	0.062	0.529	(0.271–1.003)
Surgical site					
THA	18 (24.66%)	144 (49.32%)	1.00 (reference)	–	–
TKA	55 (75.34%)	148 (50.68%)	0.001	2.445	(1.435–4.167)
Surgical type					
Primary unilateral	46 (63.01%)	247 (84.59%)	1.00 (reference)	–	–
Simultaneous bilateral	23 (31.51%)	35 (11.99%)	0.000	2.542	(1.537–4.203)
Unilateral revision	4 (5.48%)	10 (3.42%)	0.247	1.830	(0.658–5.087)
Operative time (min)	99.04 ± 35.86	98.11 ± 48.70	0.089	1.000	(0.995–1.005)
Intraoperative blood loss (mL)	228.70 ± 286.97	246.21 ± 287.47	0.676	1.000	(0.999–1.001)
TXA use					
No	21 (28.77%)	136 (46.58%)	1.00 (reference)	–	–
Yes	52 (71.23%)	156 (53.42%)	0.015	1.873	(1.127–3.110)
Drain use					
No	4 (5.48%)	164 (56.16%)	1.00 (reference)	–	–
Yes	69 (94.52%)	128 (43.84%)	0.002	4.781	(1.737–13.164)
Transfusion use					
No	40 (54.79%)	226 (77.40%)	1.00 (reference)	–	–
Yes	33 (45.21%)	66 (22.60%)	0.001	2.233	(1.405–3.550)
Human serum albumin use					
No	67 (91.78%)	267 (91.44%)	1.00 (reference)	–	–
Yes	6 (8.22%)	25 (8.56%)	0.993	0.964	(0.417–2.233)
Physical thromboprophylaxis use					
No	40 (54.79%)	100 (34.25%)	1.00 (reference)	–	–
Yes	33 (45.21%)	192 (65.75%)	0.004	0.499	(0.311–0.799)
Start time of drug anticoagulation					
Pre-operation	2 (2.74%)	34 (11.64%)	1.00 (reference)	–	–
≤ 8 h after operation	27 (36.99%)	170 (58.22%)	0.217	2.479	(0.586–10.484)
> 8 h to ≤ 12 h	18 (24.66%)	49 (16.78%)	0.035	4.844	(1.115–21.041)
> 12 h	26 (35.62%)	39 (13.36%)	0.007	7.318	(1.731–30.927)
Ambulation time					
≤ 24 h	8 (10.96%)	96 (32.88%)	1.00 (reference)	–	–
> 24 h to ≤ 48 h	8 (10.96%)	64 (21.92%)	0.425	1.494	(0.557–4.006)
> 48 h to ≤ 72 h	15 (20.55%)	82 (28.08%)	0.096	2.091	(0.878–4.981)
≥ 72 h	42 (57.53%)	50 (17.12%)	0.000	6.225	(2.89513.384)

*DVT* deep vein thrombosis, *BMI* body mass index, *ASA* American Society of Anesthesiologists, *TXA* tranexamic acid, *Hb* hemoglobin

### Univariable analysis of risk factors for DVT

The results of univariable analysis were shown in Table 1. The univariable analysis showed that the risk factors of DVT were advanced age, TKA (compared with THA), simultaneous bilateral TJA (compared with primary unilateral TJA), TXA use, drain use, transfusion, the delayed drug anticoagulation and ambulation, and no measures of physical thromboprophylaxis after surgery was associated with decreased risk of DVT.

### Multivariable analysis of risk factors for DVT

The results of multivariable analysis were shown in Table 2. And the multivariable analysis showed that the risk factors associated with significantly increased risk for DVT were TKA (compared with THA; OR, 1.836; 95% CI, 1.047–3.222; *P* = 0.034), advanced age (> 70 years compared with ≤ 60 years; OR 1.991; 95% CI 1.007–3.683; *P* = 0.028), drain use (compared with no use; OR 4.920; 95% CI 1.732–14.050; *P* = 0.003), delayed ambulation after surgery (≥ 72 h after operation; OR 6.166; 95% CI 2.859–13.301; *P* < 0.001).

**Table 2:** Multivariable analysis of independent risk factors for DVT

Perioperative factors	*P* value	OR	95% CI
Surgical site			
THA	1.00 (reference)	–	–
TKA	0.034	1.836	(1.047–3.222)
Age (years)			
≤ 60	1.00 (reference)	–	–
> 60 to ≤ 70	0.614	1.178	(0.624–2.223)
> 70	0.028	1.991	(1.007–3.683)
Drain use			
No	1.00 (reference)	–	–
Yes	0.003	4.920	(1.732–14.050)
Ambulation time			
≤ 24 h	1.00 (reference)	–	–
> 24 h to ≤ 48 h	0.310	1.669	(0.620–4.495)
> 48 h to ≤ 72 h	0.083	2.174	(0.904–5.229)
≥ 72 h	0.000	6.166	(2.859–13.301)

*DVT* deep vein thrombosis

## Discussion

This is a nested case-control study based on the large dataset of 15,326 patients underwent TJA. And to the best of our knowledge, this study included the largest Chinese patients, which was carried out to explore the risk factors of DVT postoperatively on the patients undergoing TKA and THA. We found that the occurrence rate of DVT on the patients after TKA and THA was relatively low (0.48%). And the risk factors of DVT included TKA (compared with THA), advanced age, drain use, and delayed ambulation postoperatively.

The patients undergoing TJA are thought to be at high risk for VTE postoperatively. The generation of VTE is related to three factors, which includes venous stasis, increased coagulability of the blood, and the damage of the vessel wall. VTE are consisted of PTE, calf muscle vein thrombosis, and DVT, while DVT is significantly associated with PTE [7]. Calf muscle vein thrombosis is a quite common occurrence on the patients undergoing TKA and THA, and previous studies [8, 9] have reported that the occurrence of calf muscle vein thrombosis was close to approximately 50% for the patients undergoing TJA who do not receive any form of anticoagulation. But the occurrence rate of DVT was low relatively, and a previous study [10] utilizing the National Inpatient Sample data from America containing 1,721,806 subjects revealed that the occurrence rate of DVT was 0.59% (10,197 patients) on the patients undergoing TJA. Nonetheless, the happening of DVT dramatically increased the in-hospital mortality after TJA [3].

Although many risk factors have been reported to increase the incidence of DVT after TJA, including increasing age, gender (female), obesity, diabetes mellitus, congestive heart failure, a history of VTE, cancer, and immobilization, and it was still a challenge for joint surgeons to avoid the happening of DVT after TJA [6, 11, 12]. A systematic review [13] included 44,844 cases provided by 47 studies showed that rates of symptomatic DVT were 0.63% for total or partial knee arthroplasty and 0.26% for total or partial hip arthroplasty. And our study showed that the occurrence rate of DVT in TKA was higher than THA for the Chinese patients (0.71% for TKA and 0.24% for THA; OR 1.836; 95% CI 1.047–3.222; *P* = 0.034), which is in agreement with previous study [14]. Besides, our study showed that the occurrence rate of DVT for the patients undergoing TKA and THA was low. Furthermore, previous studies [15–18] based on Asians including Singaporeans, South Koreans, and Chinese reported that the occurrence rate of DVT was lower than western population. Similarly, low rate is also found in Asians living in the West [19]. Difference in the genetic characteristic and dietary habits (vegetarian diet) have been considered as main causes of the low occurrence of DVT in Asians [20]. Furthermore, as Park et al. [21] and Pearse et al. [22] reported, the occurrence rates of DVT were 0.56% and 0.82% respectively for the patients undergoing TKA without chemoprophylaxis in Asians. So, despite that there are sufficient evidences in the Western studies to advocate routine chemoprophylaxis for the patients undergoing TJA, an individualized and more efficient risk stratification protocols for Asians may need to be developed to ensure that the patients undergoing TJA can receive more appropriate anticoagulation. However, it needs high-quality and large-sample studies to conform.

With regard to the patient-related factors, we identified that advanced age (> 70 years) is a risk factor of DVT for the patients undergoing TJA. This was in line with the findings of previous studies [3, 4]. Similarly, Suzuki et al. [23] analyzed 1232 woman from Japan undergoing obstetrical or gynecological surgery and reported that increased age (≥ 55 years) was a risk factor for perioperative VTE. Besides, although some previous studies [3, 24] revealed that the higher BMI was associated with the risk of DVT on the patients undergoing TJA, our study failed to identify it. What the reason may be was the BMI of Chinese population have a relatively narrow distribution compared with western population, which means our data was insufficient for extreme BMI values (such as BMI > 30 kg/m^2^) [25].

With respect to the surgery-related factors, we identified that delayed ambulation after surgery (≥ 72 h) and drain use were strong risk factors of DVT on the patients undergoing TJA postoperatively. Numerous previous studies [24, 26] have shown that immobilization and inactivity after operation are risk factors of DVT. What the reason may be that the ambulation later than 72 h after surgery causes blood stasis [11], which is one of main factor of DVT. Furthermore, Pearse et al. [22] reported that early mobilization protocol (beginning to walk less than 24 h after knee replacement) not only resulted in a 30-fold reduction in the risk of postoperative DVT after TKA when other risk factors were adjusted, but also brought about fewer episodes of syncope, less pain, and the need of analgesic, which may be related to psychological preparation. In short, early mobilization and ambulation after TJA were recommended because it requires no additional costly equipment, has a high level of patient acceptance, and a low incidence of associated morbidity. What the reason may be that the patients’ interpretation and understanding for their bodily symptoms can influence their behavior [27]. Although previous studies [28, 29] have showed that drain use has nothing to do with the increase of DVT after TJA, our study showed that drain use after TJA is associated with significantly increased risk for DVT after TJA, what the reason may be that the drain use may restrict mobilization of the patients postoperatively. Further, numerous studies [30, 31] showed that the drain use is associated with a higher transfusion rate and a longer postoperative length of stay. Despite high-quality and large-sample studies are needed to clarify the relationship between the drain use and the DVT for the patients undergoing TJA, the routine use of drain was not recommended in primary unilateral TJA.

There are, however, several limitations to this study. First, the patients with DVT are few relatively, so the risk factors of DVT were not identified for TKA and THA respectively. Second, although some studies [32] have revealed that the incidence of DVT following TKA was significantly lower in RA patients than in those with OA, our study failed to identify it due to the diagnoses of our cases recruited into the study were multiple. Third, the patients in the current database with extreme BMI (BMI > 30 kg/m^2^) and ASA class (especially for ASA III, IV, and V) were small. Fourth, the information of the patients was input into the system during about 2 months after patients discharged, and the follow-up time of the patients in this study was short; hence, the patients who happened DVT after data entry may be failed to be recorded, which may decreased the numbers of patients with DVT to some extent. In the context of these limitations, our results have to be interpreted with caution.

## Conclusions

In conclusion, the occurrence rate of DVT on the patients undergoing TJA was low (0.48%). And the risk factors associated with DVT included TKA (compared with THA), advanced age, drain use, and delayed ambulation postoperatively. Individualized and more efficient risk stratification protocols of anticoagulation after TJA for Chinese may need to be developed in the future.

## Data Availability

Please contact author for data requests.

## Abbreviations

ASA: American Society of Anesthesiologists class
BMI: Body mass index
CI: Confidence intervals
DVT: Deep vein thrombosis
HB: Hemoglobin
OA: Osteoarthritis
OR: Odds ratios
RA: Rheumatoid arthritis
SD: Standard deviation
THA: Total hip arthroplasty
TJA: Total joint arthroplasty
TKA: Total knee arthroplasty
TXA: Tranexamic acid
